# Editorial: Rising stars in geomicrobiology: microbial life in subsurface, seep and hydrothermal ecosystems

**DOI:** 10.3389/fmicb.2026.1808625

**Published:** 2026-02-27

**Authors:** S. Emil Ruff, Ranjani Murali, Maxim Rubin-Blum, Andreas P. Teske

**Affiliations:** 1 The Marine Biological Laboratory, Woods Hole, MA, United States; 2 University of Nevada Las Vegas, Las Vegas, NV, United States; 3 University of Haifa, Haifa, Israel; 4 University of North Carolina at Chapel Hill, Chapel Hill, NC, United States

**Keywords:** extremophiles, hot springs, hydrothermal environments, methane seeps, serpentinization, subsurface microbiology, microbial ecology, deep biosphere

Life in the subsurface persists and sometimes even thrives in some of the most physically and chemically challenging environments on Earth. These systems, ranging from deeply buried marine sediments and tectonically active ridge flanks to cold seeps, hydrothermal vents, and hot springs, are united by extreme environmental conditions, strong coupling between geology and biology, and microbial metabolisms operating close to known physiological limits. This Research Topic, *Rising stars in geomicrobiology: microbial life in subsurface, seep, and hydrothermal ecosystems*, brings together nine contributions that collectively illuminate how microbial communities are structured, sustained, and transformed across these extreme environments.

The Research Topic naturally spans an environmental gradient, beginning in the deep marine subsurface. Xiao et al. examine microbial community structure and metabolic potential in sediments from five hadal trenches, offering a rare comparative view of life under extreme pressure and chronic energy limitation. Their work highlights both shared functional traits across trench systems and localized adaptations, underscoring the importance of regional geochemistry in shaping deep biosphere communities. Complementing this broad perspective, Lever et al. focus on buried ridge flank sediments, resolving the zonation and activity of methane-cycling archaea across depth and geochemical gradients. Together, these studies establish a foundation for understanding how deeply buried microbial ecosystems persist over geological timescales.

Moving up the gradient of fluid flux and energy availability, cold seeps represent environments where subsurface processes directly intersect with the ocean. Wu et al. demonstrate that horizontal heterogeneity within cold seep sediments plays a decisive role in microbial community assembly and species coexistence. Rather than being structured solely by vertical redox zonation, seep communities respond strongly to fine-scale variations in substrate availability and physical properties. This finding reinforces the idea that spatial complexity is a key driver of diversity in chemosynthetically fueled ecosystems.

Hydrothermal systems form a central pillar of the Research Topic, with multiple contributions highlighting both methodological advances and biogeochemical consequences of hydrothermal circulation. In Guaymas Basin, Hinkle et al. apply synthetic long-read sequencing to reveal greater bacterial diversity in hydrothermal sediments than previously observed, demonstrating the power of emerging sequencing approaches to resolve microdiversity in systems shaped by thermal alteration and petroleum-derived substrates. Building on this ecological framework, Rochelle-Bates et al. quantify hydrothermal ammonium mobilization from Guaymas Basin sediments and evaluate its implications for the surrounding marine biosphere. Their results emphasize that hydrothermal systems are not only biodiversity hotspots, but also significant sources of bioavailable nutrients with basin-scale consequences. Shallow-water hydrothermal vents provide a contrasting perspective on hydrothermalism at the interface of benthic and pelagic processes. Pelliciari Silva et al. show that hydrodynamic flow and benthic boundary layer interactions strongly influence microbial community composition at Milos vents. Their work highlights the role of physical forcing in modulating microbial distributions, linking fluid dynamics to ecological outcomes in a way that is often overlooked in deeper, less accessible systems.

The Research Topic continues with continental subsurface and surface-connected analogs, extending geomicrobiological insights beyond the marine realm. Atencio et al. use site-specific incubations to explore biofilm diversity and functional adaptation in deep, ancient desert aquifers. The authors show that even long-isolated continental subsurface systems harbor metabolically versatile communities capable of responding to environmental change. Collins et al. focus on a continental serpentinizing system, where lipid biomarker evidence reveals a dominance of aerobic methanotrophy. This finding challenges traditional assumptions about methane cycling in serpentinization-driven environments and underscores the value of biomarker approaches for reconstructing *in situ* metabolisms. Finally, Howells et al. investigate methanotrophy in Yellowstone hot springs, pushing known thermal limits of microbial methane oxidation and providing key insights into how methane cycling persists under extreme temperature stress.

Taken together, the contributions in this Research Topic emphasize several unifying themes. First, fine-scale geochemical and physical heterogeneity strongly shapes microbial life in subsurface, seep, and hydrothermal environments, even where bulk conditions appear uniform. Second, advances in methodology, including long-read sequencing, targeted incubations, and lipid biomarker analyses, are transforming our ability to resolve diversity and function in these challenging systems. Third, these environments play outsized roles in global biogeochemical cycles, particularly for nitrogen and carbon, linking localized subsurface processes to broader Earth system dynamics. As part of the *Rising Stars* Research Topic, this Research Topic also highlights the creativity and interdisciplinary reach of early-career geomicrobiologists. By integrating microbiology, geochemistry, hydrology, and molecular biology across a wide range of environments, these studies reframe subsurface and fluid-driven ecosystems as integral components of the planet's interconnected biosphere, rather than isolated extremes.

